# Morphological variation and genetic diversity of *Circinaria contorta* (Megasporaceae, Ascomycota), including *C. contorta* subsp. *albida* subsp. nov.

**DOI:** 10.1038/s41598-025-04627-8

**Published:** 2025-06-04

**Authors:** Katarzyna Szczepańska, Beata Guzow-Krzemińska, Justyna Sołtysiak, Jacek Urbaniak

**Affiliations:** 1 Department of Botany and Plant Ecology, Wrocław University of Environmental and Life Sciences, pl. Grunwaldzki 24a, Wrocław, PL-50-363 Poland; 2 Department of Plant Taxonomy and Nature Conservation, Faculty of Biology, University of Gdańsk, Wita Stwosza 59, Gdańsk, PL-80-308 Poland

**Keywords:** Lichenized fungi, Megasporaceae, Phylogeny, Species diversity, Taxonomy, Typification, Biodiversity, Phylogenetics, Taxonomy

## Abstract

*Circinaria contorta* is a widely distributed, crustose, saxicolous lichen, which thallus is composed of olive-brown, white pruinose areoles and crater-like apothecia. Although it is one of the more common taxa occurring on limestone substrata in Europe and a generic type species, no type specimen has ever been designated for this species, and there are only a very few studies concerning this taxon. During research on *C. contorta* in Poland, tree main phenotypes (groups A, B and C) were delineated within the sampled material. Molecular analyses of sequences of the genus *Circinaria* from worldwide sampling indicated that representatives of *C. contorta* did not form one clade but rather were distributed at different points within the phylogenetic tree, probably due to difficulties in the correct identification of this species. Analyses based on three molecular markers, namely nucITS rDNA, MCM7, and mtSSU rDNA sequences, showed that specimens representing the phenotypic groups A and C were intermixed on the phylogenetic tree within the *C. contorta* group. However, the specimens from group B formed a separate, well-supported clade and are described herein as a new taxon at subspecies rank, *Circinaria contorta* subsp. *albida.* Specimens of group C had phenotypic features similar to those of *Circinaria hoffmanniana*, suggesting that the taxonomic concept of this species needs clarification. Furthermore, the species concept of *Circinaria contorta* subsp. *contorta* has been specified by designating an epitype specimen collected in Germany. This specimen belonged to phenotypic group A and contained aspicilin as a secondary metabolite.

**Supplementary Information:**

The online version contains supplementary material available at 10.1038/s41598-025-04627-8.

## Introduction

*Circinaria* Link is one of the genera that were resurrected^1^ after the phylogenetic revision of *Aspicilia* A. Massal. and the family Megasporaceae Lumbsch in Feige & Schmitz. In their work, the authors concluded that the family is monophyletic and nested within the Pertusariales M. Choisy ex D. Hawksw. & O. E. Erikss., a conclusion that was consistent with previous studies^2,3^. Four distinct groups were recognized within the family; based on these results, the division of Megasporaceae into *Aspicilia* (A) Massal., *Circinaria* Link, *Lobothallia* (Clauzade & Cl. Roux) Hafellner, *Megaspora* (Clauzade & Cl. Roux) Hafellner & V. Wirth and *Sagedia* Ach. was proposed^1^. During the following years, several other genera were distinguished or reinstated in the Megasporaceae, including *Antidea* T. (B) Wheeler, *Aspiciliella* M. Choisy in Werner, *Atrostelia* Paukov, Davydov & Yakovchenko, *Oxneriaria* S. Y. Kondr. & Lőkös, and *Teuvoa* Sohrabi & S. D. Leav., while others (*Agrestia* J. W. Thomson, *Chlorangium* Link and *Sphaerothallia* Nees ex Eversm.) have been withdrawn as synonyms of *Circinaria*^4^. Despite numerous studies, this family is still considered very difficult, and the concepts of many species within the family remain unclear^5,6^.

At present, *Circinaria* contains ca. 50 taxa, most of which were previously considered as members of *Aspicilia*, including *Circinaria contorta* (Hoffm.) A. Nordin, Savić & Tibell, formerly *Aspicilia contorta* (Hoffm.) Körb. Both genera, *Aspicilia* and *Circinaria*, appear to be morphologically congruent and difficult to identify; nevertheless, the two genera have several distinctive features. Most species of crustose *Circinaria* have short conidia (6–12 μm) and large (18–36 × 12–26 μm), globose or broadly ellipsoid spores, the number of which is reduced from eight to six, four or two per ascus. In contrast, the characteristic features of *Aspicilia* are longer conidia (11–40 μm) and smaller (10–27 × 8–19 μm), ellipsoid spores, typically eight per ascus^1^. In addition, *Circinaria* is the only group whose representatives may contain aspicilin as a secondary metabolite. Most representatives of the genus are distributed worldwide and occur in temperate or arid climates.

*Circinaria*, as presently circumscribed now, is a heteromorphic genus, including saxicolous crustose taxa, as well as subfruticose and subfoliose vagrant lichens (“manna lichens”), that are obligatorily unattached to the substrate^4^. Furthermore, according to phylogenetic studies^4^, the genus can be divided into two main groups—crustose and sphaerothallioid respectively. Representatives of the sphaerothallioid group are characterized by the presence of pseudocyphellae, large conidia (up to 35 μm), a well-developed cortex, a thick medulla, and a lack of secondary metabolites^4^.

*Circinaria contorta* is the generic type species and it belongs to the saxicolous, crustose group^4^. The species presents typical features of the Megasporaceae family, such as asci with a non-amyloid tholus, the ‘*Caesiocinerea*-green’ pigment^7^ present in the epihymenium, simple and hyaline ascospores and branched, anastomosing paraphysoids^8^. Typical specimens possess a crustose thallus consisting of separate or partly aggregated, olive-brown, white pruinose areoles and crater-like apothecia immersed in the areoles^9–13^. They prefer natural calcareous rocks (limestones, dolomite), as well as other base-rich substrata (brick, mortar, concrete), in sunny and warm places.

Although the phenotypic characteristics of the species appear to be defined at present, during studies concerning the ecology and distribution of *C. contorta* in Poland we have observed morphological and chemical differentiation among analysed samples. To determine the reasons for this phenotypic variation, molecular and phylogenetic analyses were conducted to examine a potential correlation between phenotypic characters and genetic variability, as well as to explore eventual cryptic speciation within the studied taxon. We used three markers, nucITS rDNA, MCM7, and mtSSU rDNA, to investigate genetic variation between newly generated sequences from samples collected in Europe (Estonia, Germany, Poland, and Ukraine) and others originating from different geographical regions (Austria, Finland, Greece, Sweden, Turkey, and the USA). Since no type specimen was designated by Hoffmann^14^ for *Verrucaria contorta* Hoffm., the basionym of *C. contorta*, and the lectotype is one of Hoffman’s illustrations, an epitypification of the epithet is proposed in this treatment. This will provide a clear definition of the phenotypic features of the taxon, and thus allow proper identification of this species.

## Results

**Fig. 1:**
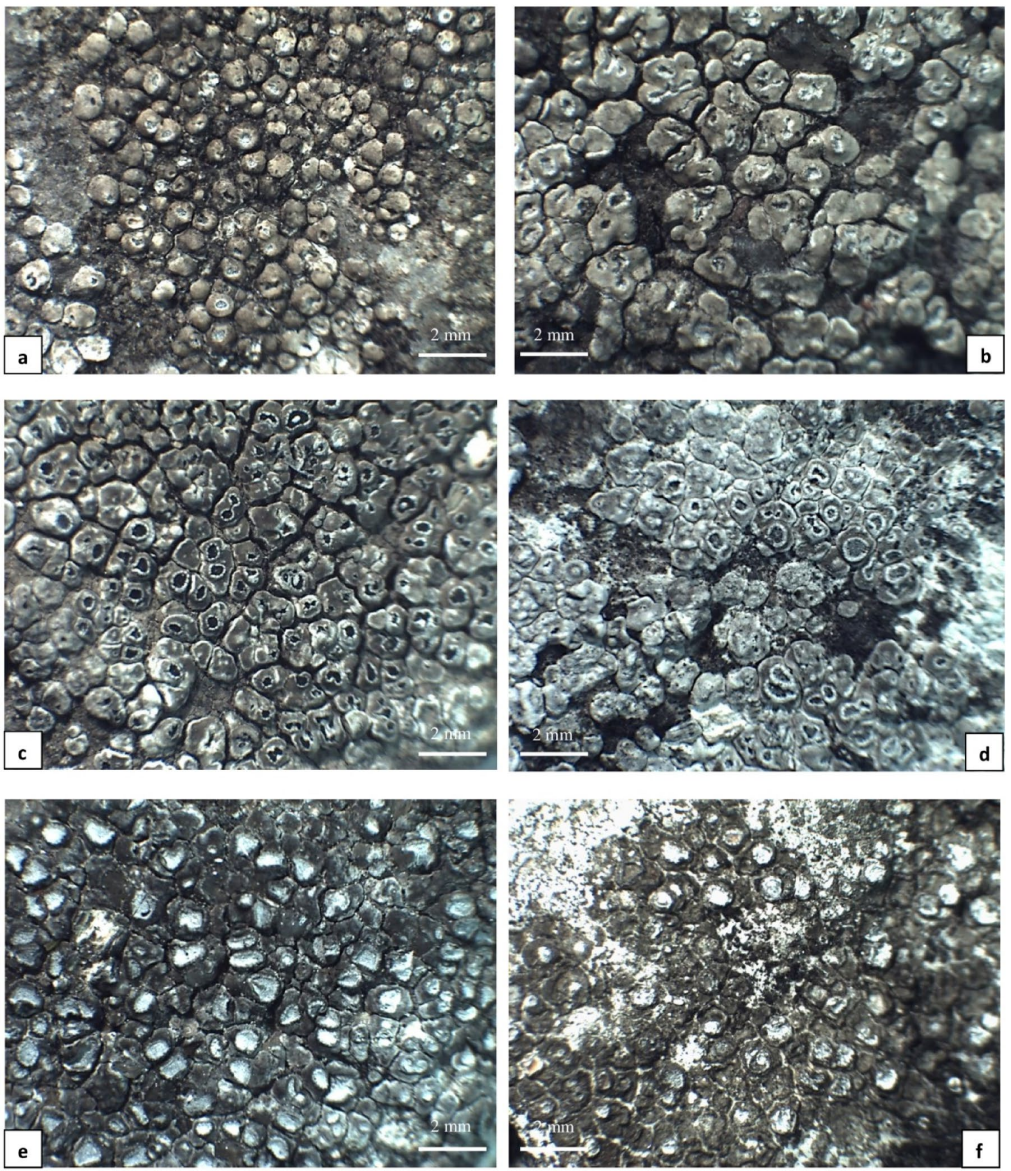
*Circinaria contorta* specimens studied; morphotypes from group A: **a** Poland (KRA-428, isolate *C. contorta* 54), **b** Poland (UGDA L-11404, isolate *C. contorta* 78), **c** Poland (KRAM L-64316, isolate *C. contorta* 17), **d** Poland (UGDA L-11514, isolate *C. contorta* 80); morphotypes from group C: **e** Poland (UGDA L-13404, isolate *C. contorta* 81), **f** Poland (WRSL, isolate *C. contorta* 217).

*Phenotypic analyses of Circinaria contorta specimens* During morphological and anatomical studies several differences were found within the studied material, that allowed us to distinguish three morphological groups. The first group was marked as “group A” (Fig. 1a–d) and included samples with convex, rounded to angular, olive-grey to olive-brown, matt, and slightly white pruinose areoles [(0.5–)0.8–1.2(–1.8) mm diam.] that were crowded in the centre and usually dispersed at the thallus margin. The apothecia are very frequent, immersed within areoles (0.2–1.2 mm diam.), crater-like, with thin and radially cracked on the inside edge, thalline margin. The discs are rounded to irregular and white pruinose. The hymenium is colourless (120–180 μm in height), with submoniliform paraphyses and an olive-brown epihymenium. Asci are 4–6-spored, with subglobose ascospores [(15–)18–25(–28) µm diam., *n* = 125]. Pycnidia occur rarely and contain filiform conidia (7–11 × 1 μm, *n* = 56). All the analysed samples of group A contained aspicilin as a secondary metabolite. This group included the largest number of examined specimens, and the group is widely distributed throughout Poland, occurring both on natural calcareous rocks and on artificial, base-rich substrata.

**Fig. 2:**
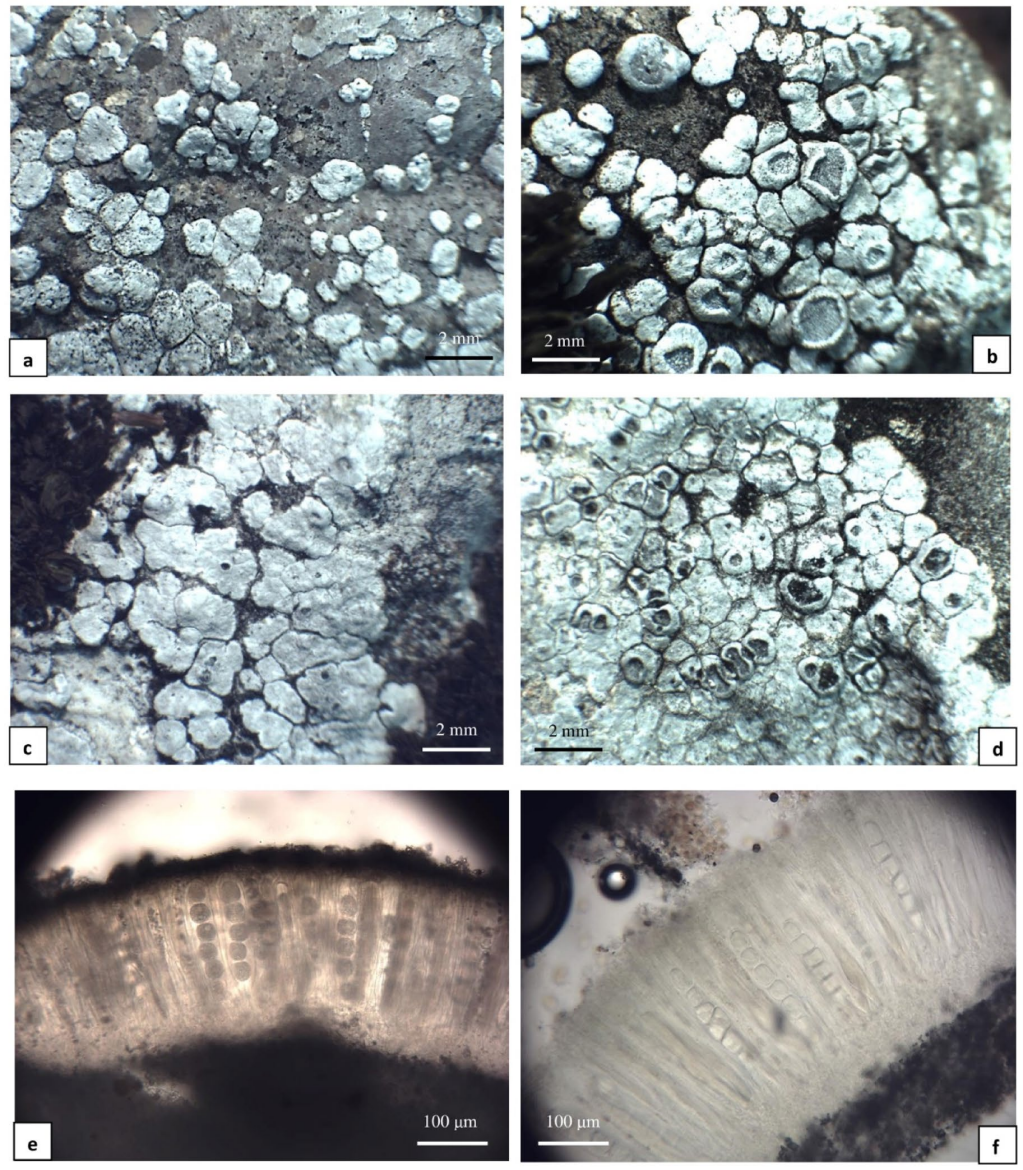
*Circinaria contorta* subsp. *albida* subsp. nov. specimens studied; morphotypes from group B: **a**, **b** Poland (WRSL, isolate *C. contorta* 196), **c** Poland (WRSL, isolate *C. contorta* 211), **d** Poland (WRSL, isolate *C. contorta* 213); **e** Apothecium section. Poland (WRSL, isolate *C. contorta* 189); **f** Apothecium section after HNO_3_ treatment. Poland (WRSL, isolate *C. contorta* 220).

The second group was designated as “group B” (Fig. 2a–f). This group represents samples with flat, rounded to angular, chalky white and densely pruinose areoles (0.5–2.5 mm diam.), partially aggregated in groups or singly dispersed. Apothecia are not always developed and are immersed in the juvenile stage but sessile and distinctly lecanorine when older (0.5–2.0 mm diam.). The thalline margins of apothecia are thin initially, then distinct, thick, raised and densely white pruinose. Discs are rounded to irregular and white pruinose. The hymenium is colourless (120–200 μm in height), with submoniliform paraphyses and an olive-brown epihymenium. Asci are 4–6-spored with subglobose ascospores [(20–)24–28(–30) µm diam., *n* = 70]. Pycnidia are uncommon and produce short conidia (4–8 × 1 μm, *n* = 16). Aspicilin was detected within all of the analysed samples marked as group B. Specimens of this group were collected from warm and sunny habitats on the Wyżyna Częstochowska Upland and Pieniny Mountains, areas of natural limestone rocks occurring in Poland. None of the specimens were found on artificial substrata.

The third group is described herein as “group C” (Fig. 1e, f). These consist of specimens with a moderately dark thallus divided into olive-brown to grey-brown, matt, and not pruinose areoles (0.4–1.2 mm diam.). The areoles are angular, flat to slightly convex, with slightly raised margins. The areoles are crowded in the centre and rarely dispersed at the thallus margin. The apothecia are common, sessile or rarely immersed (0.4–0.8 mm diam.). The thalline margin is thin to quite thick, radially cracked and white pruinose. The discs are rounded to irregular and also white pruinose. The hymenium is colourless (130–160 μm in height) with submoniliform paraphyses and an olive-brown epihymenium. Asci contain 4 to 6 subglobose ascospores [(18–)22–24(–26) µm diam., *n* = 48]. Pycnidia were not found. No chemical substances were detected in the majority of the analysed material, with aspicilin rarely observed. Specimens of this group were collected from natural limestone rocks and on artificial, base-rich substrata in different regions of Poland.

**Fig. 3:**
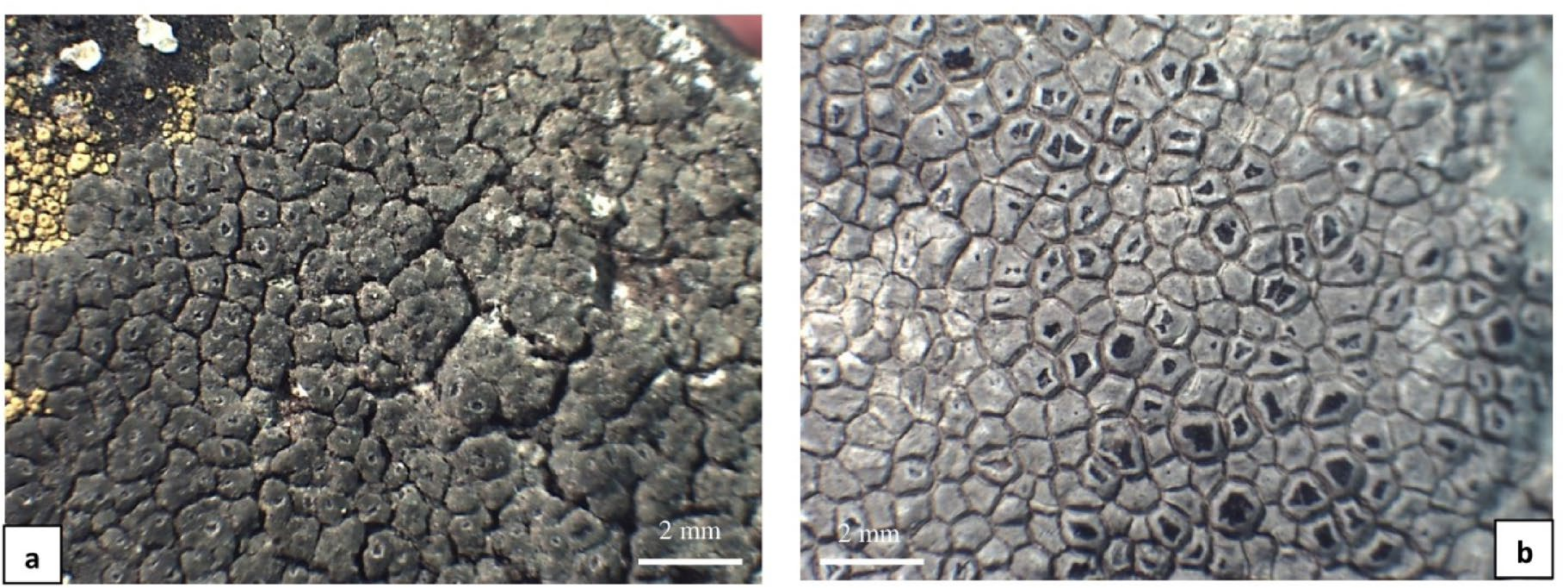
*Circinaria contorta* specimens studied; morphotypes from group X: **a** Poland (WRSL, isolate *C. contorta* 230), **b** Ukraine (KRAM L-49450, isolate *C. contorta* 37).

In addition to the above three groups of morphotypes whose features were relatively uniform, there were a few specimens that did not form a homogeneous group, with diverse phenotypic characteristics (marked as group X). These included, among others, specimens with a continuous, areolate, olive-grey or white-grey thallus (Fig. 3a, b), as well as specimens with a thallus composed of thick, grey-brown squamules. Therefore, it was impossible to assign them to any of the three aforementioned morphotypes. The results of morphological and chemical analyses of all specimens used in the study are summarized in the Supplementary Table S1 online.

**Fig. 4:**
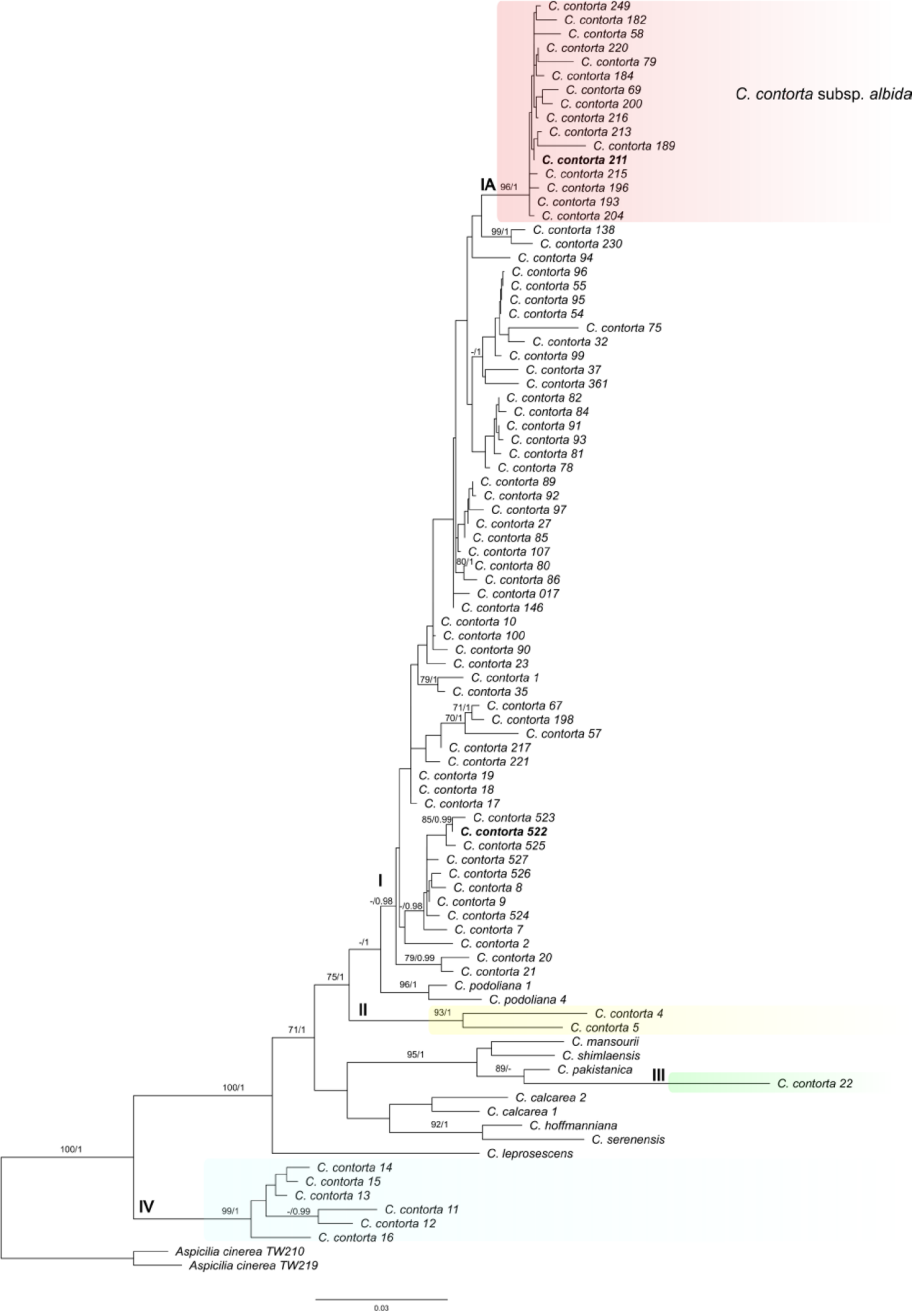
The maximum likelihood tree based on the concatenated dataset (including aligned nucITS rDNA, mtSSU rDNA and MCM7 markers) from different *Circinaria* spp. Two representatives of *Aspicilia cinerea* (L.) Körb. were used as outgroup taxa. Bootstrap support values from IQ-tree analysis (≥ 70) and PP values from Bayesian analysis (≥ 0.95) are given near the branches on the phylogenetic tree. The names of species are followed by their sample numbers. Type and epitype specimens are marked in bold. Clades/lineages I, IA, II, III and IV are indicated on the branches. Representatives of unknown species labelled as *Circinaria contorta* are marked in yellow, green, or blue. Subclade IA with representatives of *Circinaria contorta* subsp. *albida* is marked in red.

*Phylogenetic analyses* Phylogenetic analysis based on nucITS rDNA markers showed that most specimens labelled as *C. contorta* and collected in European countries (Austria, Estonia, Finland, Germany, Poland, Sweden, and Ukraine) and one from the USA (*C. contorta* 23) formed a strongly supported monophyletic clade I, closely related to *C. hoffmanniana* (S. Ekman & Fröberg ex R. Sant.) A. Nordin, *C. serenensis* (Cl. Roux & M. Bertrand) A. Nordin, and *C. podoliana* Szczepańska, Rodr. Flakus, Urbaniak & Śliwa (Fig. 4, Supplementary Fig. S1 online). However, some specimens labelled as *C. contorta* were found outside this clade. Clade II consisted of two specimens from Greece (*C. contorta* 4 and 5) from which only nucITS rDNA data were available, but this lineage was highly supported in both IQ-tree and Bayesian analyses; these specimens presumably represent a different taxon or even two taxa. As we did not have access to these specimens, and the sequences did not cluster with other species, we could not resolve their taxonomic affinity. Another specimen labelled as *C. contorta* 22 (lineage III) originated from Turkey and it also represented another unknown lineage which seems to be related to *C. mansourii* (Sohrabi) Sohrabi, *C. pakistanica* Fayyaz, M. S. Iqbal, Afshan & Khalid, and *C. shimlaensis* A. Noor, Saba & W. Akram. Moreover, specimens collected in the USA (clade IV) did not represent *Circinaria contorta.* This clade is related to *Aspicilia cyanescens* Owe-Larss. & A. Nordin and other species belonging to this genus (Supplementary Fig. S1 online). Therefore, these specimens probably belong to the genus *Aspicilia*. However, more detailed analyses of these specimens are required; this was beyond the scope of the present study.

Representatives of *Circinaria contorta* resolved in clade I, in our opinion, represent *Circinaria contorta* subsp. *contorta*, and consequently, we propose as an epitype (see Taxonomy) a specimen that was nested within this clade. However, this clade included intermingled specimens with different morphologies. We decided to recognize one highly supported lineage (subclade IA) at the subspecies level (see Taxonomy for *C. contorta* subsp. *albida*), as it comprised specimens with similar morphology (see Phenotypic analyses of *Circinaria contorta* specimens).

*Species delimitation* ABGD analysis of nucITS rDNA marker using default parameters predicted three species within the specimens of *C. contorta*. The largest group was formed by specimens from different European countries (Austria, Estonia, Finland, Germany, Greece, Poland, Sweden, and Ukraine) and one from the USA, while the second consisted of specimens collected in the USA (Clade IV), and the third was represented by a single specimen from Turkey (*C. contorta* 22 – lineage III). This delimitation was also supported by analysis of mtSSU rDNA, showing two putative species (one represented by *C. contorta* 16 collected in the USA and the second represented by specimens from clade I). ABGD analysis based on the MCM7 marker showed a single group, and in this case, specimen *C. contorta* 16 was grouped with sequences from clade I; however, this molecular marker was less variable than the nucITS rDNA and mtSSU rDNA markers.

Additional ABGD analysis of nucITS rDNA data using a gap width of 1.0 and based exclusively on sequences of *C. contorta* from clade I yielded initial and recursive partitions ranging from 1 to 50 OTUs. However, none of these partitions grouped specimens with similar morphologies analysed in this study, except specimens from clade IA that were usually treated as a single OTU.

**Fig. 5:**
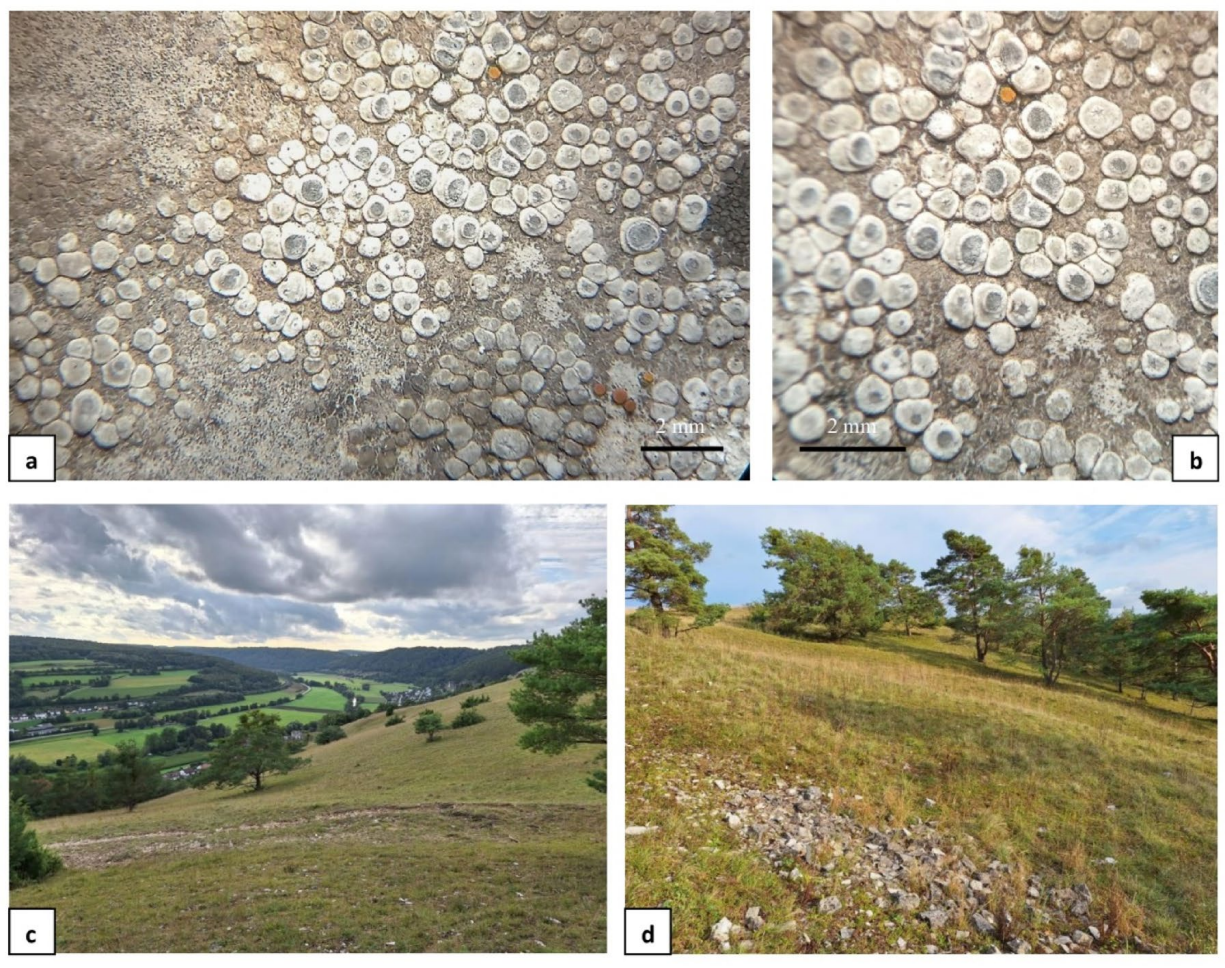
Epitype for *Circinaria contorta* subsp. *contorta*: **a**, **b** Thallus and apothecia (WRSL, isolate *C. contorta* 522); **c**, **d** Habitat - xerothermic grassland on south slopes of Altmühl river valley (Germany).

*Taxonomy* *Circinaria contorta* (Hoffm.) A. Nordin, Savic & Tibell subsp. *contorta* (Fig. 5a, b) Mycologia 102(6), 1341 (2010). ≡ *Verrucaria contorta* Hoffm., Descr. Adumb. Plant. Lich. 1(4), 97 (1790). ≡ *Lecanora contorta* (Hoffm.) J. Steiner. Verh. Kaiserl.-Königl. zool.-bot. Ges. (Wien) 65, 199 (1915). ≡ *Aspicilia contorta* (Hoffm.) Körb., Syst. lich. germ. (Breslau) 166, (1855). *Type*: (lectotype (as iconolectotype) designated by Ekman & Fröberg 1988: 215). Hoffm. Descr. Adumb. Plant. Lich. 2, T22: 4 (1790). *Epitype*: (designated here) Germany, Bavaria, the southern slopes of the Altmühl river valley, above Obereichstätt town, 48°53′33″N, 11°8′6″E, on limestone rocks in xerothermic grassland, 25 Aug. 2024, K. Szczepańska 1446 (WRSL; nucITS rDNA, mtSSU rDNA and MCM7 sequences GenBank PV021014, PV021058 and PV130764, MycoBank MBT 10026460).

*Description*—thallus lichenized, crustose, areolate. Areoles convex, rounded to angular, (0.2–)0.8–1.2(–1.7) mm in diam., olive-grey to olive-brown, matt, white pruinose, crowded in the centre and dispersed at the thallus margin. Apothecia immersed, 1–2 per areole, 0.2–0.8 mm in diam., crater-like, thalline margin thin, white pruinose and radially cracked on the inside edge, disc rounded to irregular, white pruinose. Hymenium colourless, 140–180 μm in height, paraphyses submoniliform (2–3 globose apical cells), epihymenium olive-brown, N + clearly green, K + orange-brown (*Caesiocinerea*-green), hypothecium colourless, 25–35 μm tall. Asci 4–6-spored, ascospores hyaline, subglobose, (15–)18–25(28) µm diam. Conidia filiform, 7–11 × 1 μm.

*Chemistry*—aspicilin.

*Ecology*—on natural calcareous rocks and on artificial, base-rich substrata, in the lowlands and lower mountain localities, in warm and sunny places.

*Distribution*—widely distributed and very common species, occurring in the Northern Hemisphere including Asia, Europe and North America; reported from Austria^15^, Belarus^16^, the Czech Republic^17^, Denmark^18^, Finland^19^, France^20^, Germany^13^, Italy^21^, Netherlands^22^, Norway^19^, Poland^23^, Portugal^24^, Russia^9^, Spain^24^, Sweden^19^, Switzerland^15^, Syria^25^, and Ukraine^26^, as well as the United States and Canada^27^. Molecularly confirmed from Austria, Estonia, Finland, Germany, Poland, Sweden, Ukraine, and the USA.

*Circinaria contorta* subsp. *albida* Szczepańska & Guzow-Krzem. subsp. nov. (Fig. 2c). *Holotype*: Poland, Wyżyna Krakowsko-Częstochowska Upland, Zegarowe Skały Rocks, 50°26′12″N, 19°41′27″E, alt. 450 m, on limestone, 4 June 2014, K. Szczepańska 1221 (WRSL;  nucITS rDNA and MCM7 sequences GenBank PV021004 and PV130754, MycoBank MB859222).

*Diagnosis*—differing from *Circinaria contorta* subsp. *contorta* in the larger chalky white, flat areoles (0.5–2.5 mm), larger apothecia (0.5–2.0 mm) and spores (24–28 μm), as well as higher hymenium (120–200 μm).

*Etymology*—the subspecific epithet refers to the densely pruinose, white areoles.

*Description*—thallus lichenized, crustose, areolate. Areoles flat, rounded to angular, chalky white and densely pruinose, 0.5–2.5 mm diam., partially aggregated in groups or singly dispersed. Apothecia immersed, older sessile and distinctly lecanorin, 1–2 per areole, 0.5–2.0 mm diam., thalline margin thin initially, then distinct and thick, raised, dense white pruinose, disc rounded to irregular, white pruinose. Hymenium colourless, 120–200 μm tall, paraphyses submoniliform (2–3 globose apical cells), epihymenium olive-brown, N + clearly green, K + orange-brown (*Caesiocinerea*-green), hypothecium colourless. Asci 4-6-spored, ascospores subglobose, (20–)24–28(–30) µm diam. Conidia 4–8 × 1 μm.

*Chemistry*—aspicilin.

*Ecology*—on natural calcareous rocks, in the lower mountain localities, in warm and sunny places.

*Distribution*—the area of ​​natural limestone rocks in Poland (Wyżyna Częstochowska Upland and Pieniny Mountains). Probably frequent in Europe.

*Comments*—among the European taxa of Megasporaceae growing on limestone rocks with a white pruinose thallus, the new subspecies could be confused with *Aspicilia candida* (Anzi) Hue, *A. subfarinosa* (J. Steiner) Şenkard. & Sohrabi, *Circinaria calcarea* (L.) A. Nordin, Savić & Tibell, *C. coronata* (A. Massal.) Wirth, Hauck & M. Schultz ex Paukov & Alverdiyeva, *C. serenensis* (Cl. Roux & M. Bertrand) A. Nordin, *Lobothallia farinosa* (Flörke) A. Nordin, Savić & Tibell and *Oxneriaria permutata* (Zahlbr.) S.Y. Kondr. & Lőkös. Nevertheless, *Aspicilia candida* has smaller ascospores (14–16 × 10–16 μm) and a thalline margin that shows a K + yellow reaction^13^. *A. subfarinosa* has similarly very large spores, however, they are only 4 per ascus, in addition its thallus is continuous to finely rimose, and its conidia distinctly longer (6–18 × 0.6–1 μm)^21^. *C. calcarea* also has a continuous, finely rimose, not areolate thallus, with a distinctly visible prothallus^12^, while *C. serenensis* possesses a continuous thallus without dispersed areoles at the margin, black, epruinose or rarely faintly pruinose apothecial discs, as well as longer conidia (8.5–13.5 × 1 μm)^21^. *C. coronata* differs by an endolithic to hemiendolithic, poorly visible thallus and apothecia immersed in the rock^21^. *Lobothallia farinosa* is distinguished from the new subspecies by 8-spored asci and the distinctly smaller ascospores (10–16 × 7.5–10.5 μm)^39^. *Oxneriaria permutata* also has smaller ascospores (17–20 × 9–12 μm), as well as longer conidia (16–18 × 0.5 μm), and substictic acid is present in the thallus^21^.

## Discussion

*Circinaria contorta* was described by Hoffmann in 1790 as *Verrucaria contorta*^14^. Most of Germany was indicated as a *locus classicus*. Unfortunately, no original Hoffman specimens are known; therefore, no comparative material exists. In 1988, Ekman and Fröberg^28^ published a paper extensively describing the morphological diversity observed within *C. contorta*; the authors hypothesized this to be the result of hybridization. In their work, the authors selected one of Hoffman’s illustrations as the lectotype of *Verrucaria contorta* [“Hoffm. 1790: Descript. et Adumbrat. Plant. Lich., vol. 1, T 22: 4”]. The illustration presents crustose specimens divided into small, whitish areoles aggregated in groups (“in areoles confluentes verrucae”) growing on the rock substratum. However, the image quality is insufficient for a good characterization and description of the taxon’s phenotypic characteristics. In addition, Ekman and Fröberg^28^ included a short note on the morphological features typical for *C. contorta*. In their opinion, the species possesses a crustose thallus divided into groups of white, rounded areoles (0.75–2.8 mm), more continuous and angled due to a lack of space when older, with more than one apothecium per areole and the apothecia being immersed. This description is consistent with Hoffman’s diagnosis. What is puzzling is that the authors described an N-reaction (a colourless reaction with HNO_3_) of the hymenium of *C. contorta*. Nevertheless, the lack of original material makes taxonomical and nomenclatural conclusions complicated.

Although several studies have been published concerning the taxonomy and phylogeny of the genus *Circinaria*, these were focused on the species related to “manna lichens”^4,29–35^ and only a few were dedicated to the saxicolous, crustose group^10,36–38^. Equally little information can be found on *C. contorta*, one of the most common taxa occurring on limestone rocks in Europe^12,13^, including Poland^23,39^. In the GenBank database, there was an evident lack of molecular data concerning this species, both from Europe and other parts of the world. The few sequences deposited originated from specimens collected in Austria, Finland, Greece, Sweden, Turkey, and the USA. This unexpected scarcity of molecular data makes phylogenetic analyses somewhat ambiguous and the estimation of genetic variation impossible.

*Circinaria contorta* is considered a taxon with a wide geographical distribution, occurring in the Northern Hemisphere, including North America^11,40^. Our analyses showed that most of the sequences from specimens collected from the USA clustered with species belonging to *Aspicilia*, not *Circinaria* (clade IV Fig. S1). The exception was the sequence designated as *C. contorta* 23 (voucher Knudsen 13050^41^, see Supplementary Table S2 online), the only specimen included within the *C. contorta* group (Fig. 4). Unfortunately, the source publications^41–43^ did not provide any morphological data for the samples, and thus we were unable to compare any of specimens from the USA with those from our collections at this time. However, in the literature concerning lichens occurring in North America^44^, *C. contorta* is mentioned as genetically confirmed from California, and the characteristics presented in the paper seem to be typical for this species. Nevertheless, the heterogeneous distribution of sequences on the phylogenetic tree indicates difficulties in the unambiguous identification of specimens as *C. contorta*.

A similar problem applies to the sequences *C. contorta* 4 and *C. contorta* 5 from Greece^45^ (vouchers Gavalas IRGA-473 and 479, see Table S2), that do not cluster with other sequences originated from Europe (Figs. 4 and S1), implying that taxa other than *C. contorta* may be involved. In addition, some doubts have been raised by the *C. contorta* 22 sequence from Turkey^46^ (isolate number—MGH 0.139, see Table S2), as its position on the phylogenetic tree (Figs. 4 and S1) suggests an incorrect identification of the specimen. This concern is further supported by the specimen presented in Fig. 3 of the paper^46^, which, in our opinion, does not represent this taxon. All these points suggest that the taxonomic concept of *C. contorta* remains unclear and requires clarification, including the designation of a type specimen.

During the analysis of specimens of *C. contorta* originating from Europe, we noticed some unexpected variation in morphology. Most of the specimens were included in group A (See phenotypic analysis of *Circinaria contorta* specimens). According to the descriptions in Hoffmann’s diagnosis, as well as in the other available material in the literature^9,11–13^, this phenotype seems to be the most representative for *C. contorta*. The second observed morphotype (group B) included samples with chalky white, flat, and dense pruinose areoles. In addition to the differences in the general appearance of the thallus, specimens from this group had areoles, apothecia, and ascospores larger than those of the specimens from group A, as well as a higher hymenium. The third morphotype (group C) included specimens with rather dark, olive-brown, matte and not pruinose areoles. Due to this feature of the thallus, these specimens were often labelled as *Circinaria hoffmanniana* in herbaria collections.

The morphological variation of *Circinaria contorta* has previously been observed and discussed in the literature^10,40,47,48^. In 1988, Ekman and Fröberg^28^ found the separateness of *A. contorta* and *A. hoffmanianna* at the subspecific level and decided to distinguish *Aspicilia contorta* subsp. *hoffmanniana* Ekman & Fröberg based on differences in the colour and thickness of the thallus.

According to literature data^12,28^, the most characteristic features of *A. contorta* subsp. *hoffmanniana* are a darker (grey to brownish-grey), thinner and continuous thallus, that is not divided into groups of areoles, immersed to sessile, prominent apothecia and larger ascospores^10^ (20–30 × 18–21 μm). Both taxa were later recognized as separate species—*Circinaria contorta* (Hoffm.) A. Nordin, Savić & Tibell and *Circinaria hoffmanniana* (S. Ekman & Fröberg ex R. Sant.) A. Nordin^49^. Based on our analyses, however, we conclude that the above-mentioned phenotypic features related to *C. contorta* subsp. *hoffmanniana* cannot serve as differentiating criteria. Among the studied specimens, we also observed those (group C) whose morphology would fit the description of the mentioned taxon, however; further phylogenetic analyses indicated them as *C. contorta*. Because no additional phenotypic features were described in relation to *C. hoffmanniana* after the new combination was provided, the taxonomic concept of this species, as in the case of *C. contorta* s. str., needs further clarification.

Apart from the morphology, there are also some inaccuracies regarding the content of the secondary metabolites in *C. contorta* thalli, a factor that is variously treated in literature sources. This applies to aspicilin, which is either reported as not present^12,13^ or “usually” detectable^10,11^ in the thallus. These data are not entirely consistent with our results, as most of the specimens analysed in this work contained aspicilin. The exceptions were specimens from group C with a dark and thin thallus, in which aspicilin was not always detected. However, the absence of aspicilin could be the result of too little material used in the TLC, meaning that aspicilin was not detected due to its trace quantity. Furthermore, the production of some secondary metabolites may be induced by environmental factors, and this could also cause the lack of aspicilin in some specimens. Although the chemistry of the thallus cannot be used as a main criterion for distinguishing species, it is often regarded as a feature that facilitates diagnosis^6,50–52^. Therefore, the composition of secondary metabolites should be as precise as possible. In our opinion, the presence of aspicilin should be a diagnostic feature in the case of *C. contorta.*

Despite differences in morphology, most of the newly generated sequences clustered with representatives of *C. contorta* downloaded from GenBank originating from Austria, Finland, and Sweden (clade I on Fig. 4). Although cryptic speciation is often observed within both macro- and microlichens^6,53–57^, we were unable to determine this in the case of *C. contorta*, a result that may be related to geographically limited sampling. The morphotypes of the A and C groups described in the Results section were intermixed in the phylogenetic tree and did not form distinct subclades. Moreover, specimens not assigned to group A or C and marked as the X group were also intermixed but clustered within *C. contorta*. The exception, however, was group B, where the specimens formed a separate and well-supported subclade (IA in Fig. 4 and S1), which, considering the differences in morphology, is proposed here as a new taxon at the subspecies rank.

As a result of the above analyses, we propose to designate an epitype for *Circinaria contorta* subsp. *contorta* due to the appearance of the lectotype, as this is ambiguous and cannot be interpreted critically to accurately use the name of the taxon. It is not possible to determine the morphological features of the taxon presented from Hoffmann’s illustration, and it may be interpreted as a species other than *C. contorta*. The most appropriate material for an epitype seems to be a specimen collected in Bavaria in Germany (Fig. 5a, b), as this country is mentioned in Hoffman’s description as the *locus classicus* for this taxon (“In plerisque Germaniae locis ad rupes et faxa provenit”). The specimen grew on calcareous rock in xerothermic grassland on the south slopes of the Altmühl River valley (Fig. 5c, d). It has a typical morphology described for group A and contains aspicilin as a secondary metabolite in the thallus. Moreover, the sequence obtained from the indicated specimen has been deposited in the GenBank database; it is publicly available and can be used to identify this species unequivocally. On the phylogenetic tree (Fig. 4), this sequence forms a highly supported sister clade with specimens collected in Sweden (*C. contorta* 8 and *C. contorta* 9) and identified as such by A. Nordin (see Table S2). In addition, a new subspecies of *C. contorta* represented by specimens from phenotypic group B is described herein as *Circinaria contorta* subsp. *albida.*

## Materials and methods

*Specimen sampling* The study was based on material borrowed from Polish herbaria: KRA, KRAM, KRAP, KTC, LODL, OLTC, UGDA and WRSL. Our sampling focused on specimens labelled as *Circinaria contorta*, although for comparison, some specimens marked as *C. hoffmanniana* (S. Ekman & Fröberg ex R. Sant.) A. Nordin and *C. calcarea* (L.) A. Nordin, Savić & Tibell were also analysed. A total of 69 specimens were used for the morphological, chemical, and molecular studies. All specimens were collected in Central and Eastern Europe (Estonia, Germany, Poland, and Ukraine). For the phylogenetic analyses, 57 nucITS rDNA, 42 MCM7 and 45 mtSSU rDNA newly generated sequences were used, as well as 117 sequences of the family Megasporaceae from GenBank (Table S2).

*Morphology and chemistry* The morphological and anatomical data of the *Circinaria contorta* specimens were obtained using dissecting and light microscopes following routine techniques. All specimens were assessed for morphological characters such as shape, colour, and size of thallus areolae, the shape and colour of the apothecial margin and disc, the colours of the hymenium, epihymenium, and hypothecium, the morphology of paraphyses (moniliform, submoniliform), as well as the shapes of conidia and ascospores. For light microscopy, vertical sections of apothecia were cut by hand using a razor blade and mounted in water. Hymenium (height), conidia (length) and ascospores (length and width) measurements were made in water. At least ten measurements of morphological variables and measurements of 20 spores and conidia were made for each sample, and their minimum and maximum values were calculated. The presence of the epihymenium pigment ‘*Caesiocinerea*-green’^7^ was detected by colour reaction after the application of HNO_3_ (50%).

The chemistry of the specimens was investigated by spot tests using KOH applied on the lichen medulla and thallus, as well as by thin layer chromatography (TLC) employing in A and C solvent systems using the standardised method of Culberson^58^ and following Orange et al.^59^.

*DNA extraction, PCR amplification, and DNA sequencing* Genomic DNA from analysed specimens was extracted after mechanical cell disruption using Mixer Mill MM400 (Retsch, Haan, Germany). Isolation was performed by the CTAB method according to the standard protocol described by Doyle and Doyle^60^. The quality of DNA was determined using 1% TBE agarose electrophoresis. PCR reactions were performed in a 20 µl total volume with a Taq DNA reaction buffer containing MgCl_2_, a 0.2 mM dNTP mix, 1u Taq DNA polymerase (Thermo Fisher Scientific, Waltham, MA, USA), 0.5 mM of each primer used for the markers nucITS rDNA, MCM7, and mtSSU rDNA (Table 1), and 1.0 µl of total genomic DNA. The proper annealing temperature was determined using the gradient method. The PCR programme consisted of an initial denaturation at 95 °C for 6 min, followed by 30 cycles at 95 °C for 30 s, 45 s at the annealing temperature specific for each primer set used (51.0 °C for nucITS rDNA, 51.2 °C for MCM7, and 52 °C for mtSSU rDNA), 72 °C for 45 s, with a final extension at 72 °C for 10 min. A Veriti Thermal Cycler (Life Technologies, Carlsbad, CA, USA) was used for the PCRs. Detailed information on the primer sequences used for the study is provided in the Table 1. Amplification products were separated on 1% agarose gels photographed and compared with the DNA GeneRuler 100 bp (Thermo Scientific, MA, USA). Bands corresponding to the amplified regions were excised from the agarose gels and then purified by ethanol precipitation. Clean samples were sent to a sequencing service (Macrogen, the Netherlands). All laboratory analyses were performed at the Department of Botany and Plant Ecology at the Wrocław University of Environmental and Life Sciences.

**Table 1:** Primers used for this study.

Primer name	Primer sequence	References
ITS1F ITS4	CTTGGTCATTTAGAGGAAGTAA TCCTCCGCTTATTGATATGC	Gardes and Bruns (1993)^79^ White et al. (1990)^80^
MCM7for MCM7rev	CGTCACTACAAAACAATTCACC CGCCCATCTCTTTTGTGAC	Fryday et al. (2021)^81^
mrSSU1 mrSSU2 mrSSU2R mrSSU3R	AGCAGTGAGGAATATTGGTC CTGACGTTGAAGGACGAAGG CCTTCGTCCTTCAACGTCAG ATGTGGCACGTCTATAGCCC	Zoller et al. (1999)^82^

*Sequence alignment and phylogenetic analysis* Before phylogenetic analysis, the newly obtained sequences were subjected to a BLAST search in order to check their identity^61^.

For the phylogenetic analyses we used sequences of *Circinaria contorta* specimens and additional representatives of the genera *Circinaria* and *Ascpicilia*. *Aspiciliella cupreoglauca* (B. de Lesd.) Zakeri, Divakar & Otte, *A. intermutans* (Nyl.) M. Choisy and *A. portosantana* Sipman & Zakeri were used as outgroup in the analysis of nucITS rDNA dataset. In total, the final alignment consisted of 150 sequences and 1065 cites. the SYM + I + G4 model was chosen according to the BIC. The best ML tree was inferred using IQ-TREE with 1000 ultrafast bootstrap replicates as implemented in the IQ-TREE web server^62–66^.

The independent alignments for each marker were generated in MAFFT using the auto option and the default parameters^67,68^. The datasets were then subjected to Gblocks selection of poorly aligned sites employing less stringent parameters^69^ using Seaview software^70,71^. Single-locus matrices consisted of 87 sequences for nucITS rDNA, 55 sequences for mtSSU rDNA, and 48 sequences for MCM7. The best ML tree was inferred for each locus using IQ-TREE with 1000 ultrafast bootstrap replicates as implemented in the IQ-TREE web server^62–66^.

For the final analysis, we concatenated three markers, resulting in a dataset of 91 terminals and 2004 positions. The concatenated dataset was subjected to IQ-TREE analysis to find the best-fitting nucleotide substitution models^62,64–66^ and the following nucleotide substitution models for the three predefined subsets were selected: TIM3e + R2 for nucITS rDNA, TPM2u + F + R3 for mtSSU rDNA, and K3P + R3 for the MCM7 marker. The search for the maximum likelihood tree was performed in IQ-TREE web server and followed with 100 standard bootstrap replicates^62–66^. Two representatives of *Aspicilia cinerea* (L.) Körb. were used as outgroup taxa.

Bayesian analysis was carried out using a Markov Chain Monte Carlo (MCMC) method in MrBayes v. 3.2.6^72,73^ on the CIPRES Web Portal^74^ using previously selected models. Two parallel MCMC runs were performed, each using four independent chains and ten million generations, with sampling every 1000th tree. The resulting log files were analysed using Tracer 1.7.2^75^. Posterior probabilities (PP) were determined by calculating a majority-rule consensus tree after discarding the initial 25% of the trees of each chain as a burn-in. The convergence of the chains was confirmed by the convergent diagnostic of the Potential Scale Reduction Factor (PSRF), which approached 1 and the ‘average standard deviation of split frequencies’ was < 0.01^76^.

Phylogenetic trees were visualised using FigTree v. 1.4.3^77^ and modified using Inkscape (https://inkscape.org/). Bootstrap support (BS values ≥ 70) and PP values (values ≥ 0.95) are given near the branches on the phylogenetic tree.

*Species delimitation analysis using Automatic Barcode Gap Discovery* In order to infer the most likely species numbers in our dataset, we used a method for single-locus DNA-based species delimitation, the Automatic Barcode Gap Discovery (ABGD)^78^. The analysis was performed for each locus separately using DNA matrices without outgroup taxa, and we used the Jukes-Cantor method and default parameters including Pmin = 0.001, Pmax = 0.1, steps = 10 and X = 1.5 or 1.0.

## Electronic supplementary material

Below is the link to the electronic supplementary material.

Supplementary Material 1

Supplementary Material 2

Supplementary Material 3

## Data Availability

DNA sequences are deposited in GenBank. Accession numbers are available in Supplementary Table S2 online. The DNA matrix used for phylogenetic analyses of *Circinaria*, as well as the phylogenetic tree, are available under: https://doi.org/10.6084/m9.figshare.28399367.
